# Collagen type II solution extracted from supercritical carbon dioxide decellularized porcine cartilage: regenerative efficacy on post-traumatic osteoarthritis model

**DOI:** 10.1186/s40643-024-00731-1

**Published:** 2024-02-03

**Authors:** Srinivasan Periasamy, Yun-Ju Chen, Dur-Zong Hsu, Dar-Jen Hsieh

**Affiliations:** 1 R&D Center, ACRO Biomedical Co., Ltd, 2nd. Floor, No.57, Luke 2nd. Rd., Luzhu District, Kaohsiung City, 82151 Taiwan; 2 Department of Environmental and Occupational Health, College of Medicine, National Cheng Kung University, 138 Sheng-Li Rd., Tainan, 70428 Taiwan

**Keywords:** Type II collagen solution, Supercritical carbon dioxide (scCO_2_), Decellularized porcine cartilage graft (dPCG), Medial meniscectomy (MNX), Osteoarthritis (OA)

## Abstract

Osteoarthritis (OA) of the knee is a common degenerative articular disorder and is one of the main causes of pain and functional disability. Cartilage damage is frequently linked to elevated osteoarthritis incidence. Supercritical carbon dioxide (scCO_2_) decellularized cartilage graft produced from the porcine cartilage is an ideal candidate for cartilage tissue engineering. In the present study, we derived collagen type II (Col II) solution from the scCO_2_ decellularized porcine cartilage graft (dPCG) and compared its efficacy with hyaluronic acid (HA) in the surgical medial meniscectomy (MNX) induced post-traumatic osteoarthritis (PTOA) model. Dose-dependent attenuation of the OA (12.3 ± 0.8) progression was observed in the intra‐articular administration of Col II solution (7.3 ± 1.2) which significantly decreased the MNX-induced OA symptoms similar to HA. The pain of the OA group (37.4 ± 2.7) was attenuated dose-dependently by Col II solution (45.9 ± 4.1) similar to HA (43.1 ± 3.5) as evaluated by a capacitance meter. Micro‐CT depicted a dose-dependent attenuation of articular cartilage damage by the Col II solution similar to HA treatment. A significant (*p* < 0.001) dose-dependent elevation in the bone volume was also observed in Col II solution-treated OA animals. The protective competence of Col II solution on articular cartilage damage is due to its significant (*p* < 0.001) increase in the expression of type II collagen, aggrecan and SOX‐9 similar to HA. To conclude, intra‐articular administration of type II collagen solution and HA reestablished the injured cartilage and decreased osteoarthritis progression in the experimental PTOA model.

**Graphical Abstract:**

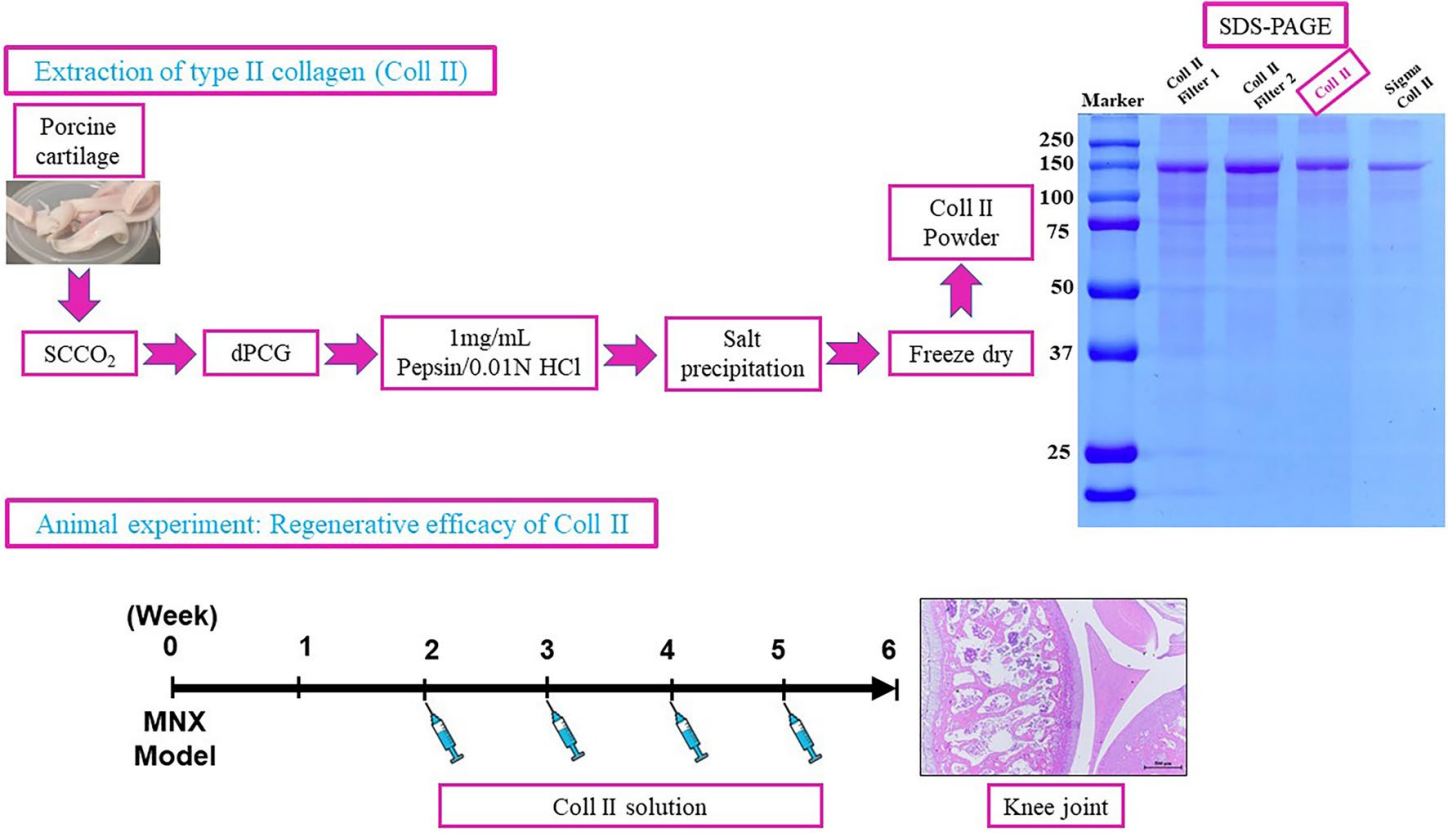

**Supplementary Information:**

The online version contains supplementary material available at 10.1186/s40643-024-00731-1.

## Introduction

Osteoarthritis (OA) is the most dominant and persistent ailment of synovial joints in humans and remains the main reason for disability around the world (Pucha et al. 2020). OA is generally manifested by the breakdown of the articular cartilage in the joints with associated clinical conditions such as subchondral bone sclerosis, formation of osteophytes, and synovial inflammation (Loeser et al. 2012; Pucha et al. 2020; Walsh et al. 2009;). About 9.6% of men and 18.0% of women over the age of 60 were affected by OA as estimated by the World Health Organization (WHO) (WHO 2016; Pucha et al. 2020). As compared to men, women tend to advance into more aggressive clinical phenotypes, related to increased pain and a substantial decrease in mobility and quality of life (Zhang et al. 2010; Pucha et al. 2020).

Pain is the principal and key clinical symptom of OA, (Davis et al. 2010) and most patients suffer from pain even after using painkillers. Augmentation of the OA pain can done by many mechanisms, within the knee joint, one therapeutic option is via attenuating the sensitization of pain. Pain is closely linked to synovitis and joint damage (Scanzello and Goldring 2012). The OA can be induced by the MNX model via the transection of the medial collateral ligament made through the meniscus. The resulting pathological alterations were comparable to the post-traumatic human OA resulting from instability and incongruity between joint surfaces (Mapp et al. 2013). Nociceptive pain is the result of weight-bearing asymmetry, equivalent to pain in human standing with OA. In MNX model OA, there is pronounced pain due to the enhanced weight-bearing asymmetry (Mapp et al. 2013). Pro-inflammatory cytokines such as interferon (IFN)-γ, interleukin (IL)-7, and IL-12 correlated significantly with the level of knee pain. The clinical relevance of specific inflammatory mediators and cell types remains elusive. However, the degradation of articular cartilage triggers inflammatory mediators leading to the development of OA-related pain via interactions between the immune and nervous system (Nees et al. 2019). Human knee OA is often associated with synovitis (Hill et al. 2007), which leads to pain and is linked to the advancement in knee joint deterioration (Dieppe et al. 2005). Cartilage damage and osteophyte formation both have been associated with synovitis in human and animal models (Dieppe et al. 2005; Mapp et al. 2013). During OA vascular growth occurs at the osteochondral junction in humans (Suri et al. 2007), recent findings showed osteochondral vascularity in rats following MNX surgery (Mapp et al. 2008, 2013).

At present, the therapeutic choices for OA include the use of painkillers, nonsteroidal anti-inflammatory drugs (NSAIDs), intra-articular (IA) corticosteroid injection, weight reduction and in end-stage OA cases surgical treatment is often needed. All therapeutic choices should be well thought out before going to surgical treatment for OA, as it is costly and risky. Viscosupplementation with intra-articular hyaluronic acid (HA) injection was approved by the Food and Drug Administration (FDA) in 1997. Viscosupplementation is a well-recognized therapeutic choice for knee OA (Migliore et al. 2010). Five injectable forms of HA approved by the United States FDA including Hyalgan^®^, Supartz^®^, Orthovisc^®^, Synvisc^®^, and Euflexxa^®^, are currently in the market as therapeutic options (Migliore et al. 2010). Type II collagen is effective in reducing arthritic pain in animal models (Gupta et al. 2009; Deparle et al. 2005; Mannelli et al. 2013) as well as in improving clinical outcomes in patients with rheumatoid arthritis (Wei et al. 2009). On comparing type II collagen to glucosamine and chondroitin in knee OA patients, treatment with type II collagen was found to be more effective in reducing OA parameters than glucosamine and chondroitin (Crowley et al. 2009).

The medial meniscus destabilization model is used to establish the development of post-traumatic OA (PTOA) functionally, structurally and biochemically. In addition, it assesses numerous biological interventions in small animal models (Sambamurthy et al. 2018; Bansal et al. 2020). In the present study, we used collagen type II solution (Col II) extracted from scCO2 decellularized porcine cartilage graft (dPCG) (Duarte et al. 2022; Han et al. 2023; Antons et al. 2018; You et al. 2018) and the therapeutic efficacy for knee cartilage regeneration is equated to commercially available therapeutic viscosupplementation hyaluronic acid in the meniscal transaction (MNX)-induced rat OA.

## Materials and methods

### Collagen type II solution production from decellularized porcine cartilage

The cartilage source was bought from Tissue Source, LLC (Lafayette, Indiana USA). Porcine cartilage graft (PCG) was decellularized using supercritical CO_2_ extraction technology to produce decellularized porcine cartilage graft (dPCG). Production, characterization and biocompatibility of dPCG were established and published (Wu et al. 2021; Hsieh et al. 2020). The dPCG was digested in 0.01N hydrochloric acid containing one milligramme per millilitre of pepsin. The supernatant was precipitated using NaCl and sodium phosphate buffer. The precipitated Col II was reconstituted in saline for intra-articular injections.

### *Characterization of Col II extracted using scCO*_*2*_* extraction technology*

We have added the characterization of the product in the manuscript (DNA, Hydroxyproline assay, SDS-PAGE). DNA quantification was done using a commercial kit (NautiaZ Tissue DNA Mini Kit, Nautiagene) genomic DNA was extracted from the final product type II collagen solution and was quantified by using a microplate reader measured at 260 nm (Wu et al. 2021). Type II collagen solution was quantified using hydroxyproline assay using a commercial kit (abcam, Hydroxyproline Assay Kit (Colorimetric) (ab222941). A standard 8% SDS-PAGE was run to analyse the type II collagen content in the production process including filter I, filter II and the final product for comparison type II collagen from Sigma was used (Hsieh and Srinivasan 2020).

### Animals

Wistar rats male (10–11 weeks, BioLASCO Taiwan Co., Ltd.) were acclimated at room temperature, 25 ℃ and humidity of 70%, 2 rats per cage. The animals were marked by tail marking for groupings. In-house autoclaved Lab Diet^®^ 5010 Rodent Diet (PMI Nutrition International, U.S.A.) and in-house autoclaved RO water were fed ad libitum*.* Experimental protocols and animal care were in compilation with institutional and international standards (Principles of Laboratory Animal Care, National Institutes of Health) and were approved (IACUC-108310) by the Institutional Animal Care and Use Committee of the National Cheng Kung University (Tainan, Taiwan). In the complete experimental study period observations were done for general conditions clinical signs, for abnormal signs and death will be recorded.

### Rat model MNX surgery for OA induction

Under the effect of 3% of isoflurane as anaesthesia, the MNX surgery was done, and the left hind limb of the rat was shaved and cleansed with povidone-iodine. The medial side skin at the knee cap was incised about 2 cm to expose the patella and patellar tendon. After moving the patellar laterally using curved forceps. The knee was flexed, to expose the joint capsule. The joint capsule was removed, and subsequently, the medial meniscus was completely removed to achieve an unstable knee joint. The patellar was aligned to its original midline position and sutured the fascia and skin sequentially using 3-0 polydioxanone and 3-0 nylon threads. The control group rats did not endure MNX surgery (O'Brien , McDougall, 2020).

### Animal groupings

**Fig. 1:**
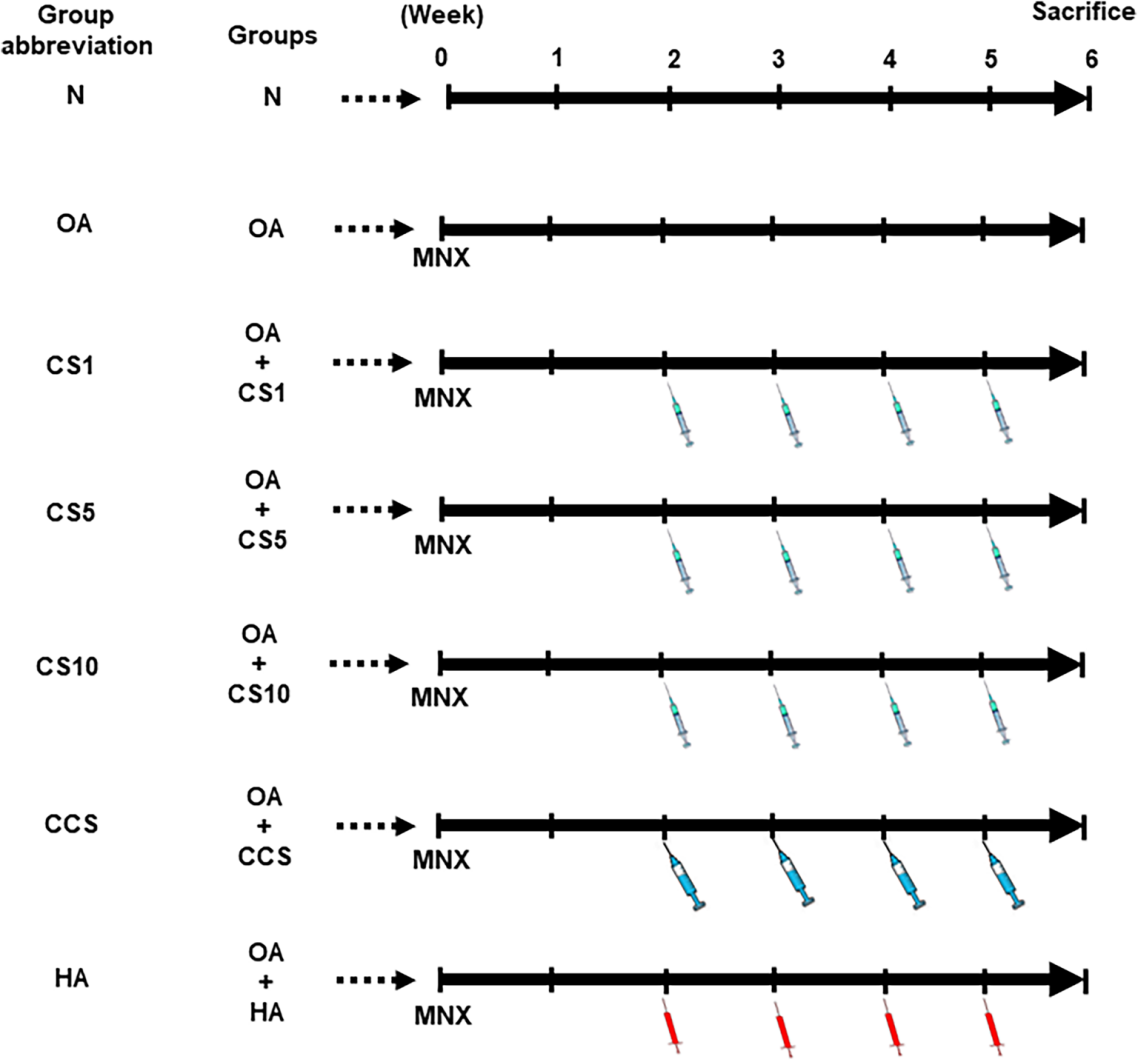
Experimental protocol of the MNX‐induced OA and the treatment schedule of different groups. The body weight changes were recorded 6 weeks after MNX surgery in rats. Data are means ± standard deviation (SD) (*n* = 6)

Animals were grouped into seven groups with six animals per group. Group I—normal (N), group II—MNX-induced OA (OA), group III—MNX-induced OA intra-articularly injected with Col II 1 mg/kg (OA + CS1), group IV—MNX-induced OA intra-articularly injected with Col II 5 mg/kg (OA + CS5), group V—MNX-induced OA intra-articularly injected with Col II 10 mg/kg (OA + CS10), group VI—MNX-induced OA intra-articularly injected with Col II 1 mg/kg + dPCG 40 mg/kg (OA + CSS), group VII—MNX-induced OA intra-articular injection with hyaluronic acid (8 mg/ml) (OA + HA). The final injectable volume for each dose is 100 µL. The animals were administered intra-articular injection with the Col II solution, dPCG, hyaluronic acid or saline once a week for 4 weeks starting from the 2nd week after MNX surgery (Fig. 1).

### Pain behaviour assessment (weight distribution in hindlimb)

An in-capacitance meter was used to evaluate the pain in the knee joint (IITC, Inc., Woodland Hills, CA, USA), the animals were made to stand only on the hind limbs which measure the weight-bearing (Philpott et al. 2017; Wu et al. 2021).

### Micro-CT examination of the experimental knee joint

The tibia metaphysis micro-CT investigation was done using the system 1076 micro-CT-40; Skyscan (Aartselaar, Belgium), equipped with a high-resolution and low-dose X-ray scanner. The functional input of the X-ray tube was done with a photon energy of 50 kV, current of 200 µA and exposure time of 1200 ms through a 0.5-mm-thick filter. The scanning parameters were set at 35-mm width and 35-µm pixels. The scanned images were recreated by a standardized practice, and the data sets for each tibia sample were resampled with software (CTAn; Skyscan) to position each sample correspondingly (Hsu et al. 2012). The rebuilding of the cross-sectional images was carried out by employing a filtered back-projection algorithm (1076 micro-CT-40; Skyscan, Aartselaar, Belgium). The cross-section images of 1800 were reconstructed for each scan, positioned middle over the knee joint, with an interslice distance of 1 pixel. The images of 1500 × 1500 pixels each, 1-pixel size, were stored as 8-bit images (Wu et al. 2021).

### Knee cartilage tissue processing

The experimental animal’s knee tissue samples were demineralized with EDTA and formic acid (Formical 2000; Decal Corporation, Congers, NY) and shaken over a rocker for 3 weeks at room temperature. The knee tissue samples were saturated in a solution of PBS with 15 wt% sucrose, followed by PBS with 30% sucrose, and embedded in wax, 6-μm-thick longitudinal sections were cut using a microtome and mounted on glass slides. Hematoxylin and eosin (H&E) and safranin-O and staining were done. Using a light microscope the sections were evaluated and images were recorded using a digital camera (Axio Imager.Z2 equipped with AxioCam MRc5; Carl Zeiss MicroImaging GmbH, Germany) (Wu et al. 2021).

### Histological scoring

The scoring was done by two independent scorers who are blinded to the study using a previously well-known scoring system for knee joint tissue histological alterations (Hawker 2016; Wu et al. 2021). The criteria for histological scoring are shown in Table 1.

**Table 1:** The histological scorings for the assessment of cartilage repair were carried out following Wu et al. (2021)

Histological description	Scoring
Chondrocyte distribution	
Columnar	3
Mixed columnar-clusters	2
Clusters	1
Individual or disorganized cells	0
Chondrocyte cellularity	
A similar number of chondrocytes	3
More chondrocytes	2
Fewer chondrocytes	1
No chondrocytes	0
Joint surface regularity	
Smooth, intact surface	3
Surface fissures (< 25% new surface thickness)	2
Deep fissures (25–99% new surface thickness)	1
Complete disruption of the new surface	0
Safranin O staining	
Similar staining intensity	4
Stronger staining intensity	3
Moderate staining intensity	2
Poor staining intensity	1
Little or no staining intensity	0

### Collagen type II, aggrecan and SOX-9 immunostaining

The tissue section mounted slides were immersed in preheated 10 mM citrate buffer (pH 6.0) at 100 ℃ for 30 min for antigen retrieval. Immunostaining was carried out employing a standard avidin–biotin-peroxidase complex detection kit (DakoCytomation, Glostrup, Denmark) as per the instructions for use. Primary antibodies were incubated for 60 min at room temperature (collagen type II ab34712 and aggrecan 1:100, ab3778, Abcam, Cambridge, MA, USA; SOX-9 1:100, 82640S, Cell signalling technology, Danvers, MA, USA) and secondary antibody Goat Anti-Rabbit IgG-Biotin, ab6720, 1:1000 dilutions, Abcam, Cambridge, MA, USA Goat Anti-Mouse IgG1-Biotin, ab98691, 1:1000 dilutions, Abcam, Cambridge, MA, USA), then added biotinylated secondary antibody and incubated for 60 min, and peroxidase-conjugated streptavidin for 30 min and developed using nickel plus DAB solution for 5–10 min to localize positive staining and counterstained with hematoxylin for the 30 s to 1 min, mounted and photographed and semi-quantified by ImageJ software (Wayne Rasband, National Institute of Health, USA).

### Statistical analysis

The statistical analysis was done with the SPSS software (SPSS 12, Chicago, IL, USA). One-way analysis of variance (ANOVA) followed by a Student’s t-test was employed to evaluate the data, respectively. *p* < 0.001, *p* < 0.01 and *p* < 0.05 was considered statistically significant.

## Results

### *Characterization of Col II extracted using scCO*_*2*_* extraction technology*

The DNA content of the Col II product produced using scCO_2_ extraction technology was found to be zero. Type II collagen content of the final products depicted 5.76 ± 0.13 mg/mL. The SDS-PAGE electrophoresis of the final product revealed type II collagen produced by using scCO_2_ extraction technology was found to contain a single α-1 band of type II collagen with the same degree of purity as Sigma standard collagen (Additional file 1: Fig. S1).

### Efficacy of Col II solution and hyaluronic acid on body weight

The body weight changes at the end of the experimental periods were 25.4 ± 1.9 (in N group), 22.4 ± 2.8 (in OA group), 24.9 ± 2.6 (in CS1 group), 26.5 ± 5.3 (in CS5 group), 27.5 ± 1.9 (in CS10 group), 24.3 ± 3.8 (in CCS group), and 26.5 ± 3.2 (in HA group), respectively. However, no significant difference was found between the experimental groups at 6 weeks after treatments (Fig. 1).

### Efficacy of Col II solution and hyaluronic acid on pain behaviour

**Fig. 2:**
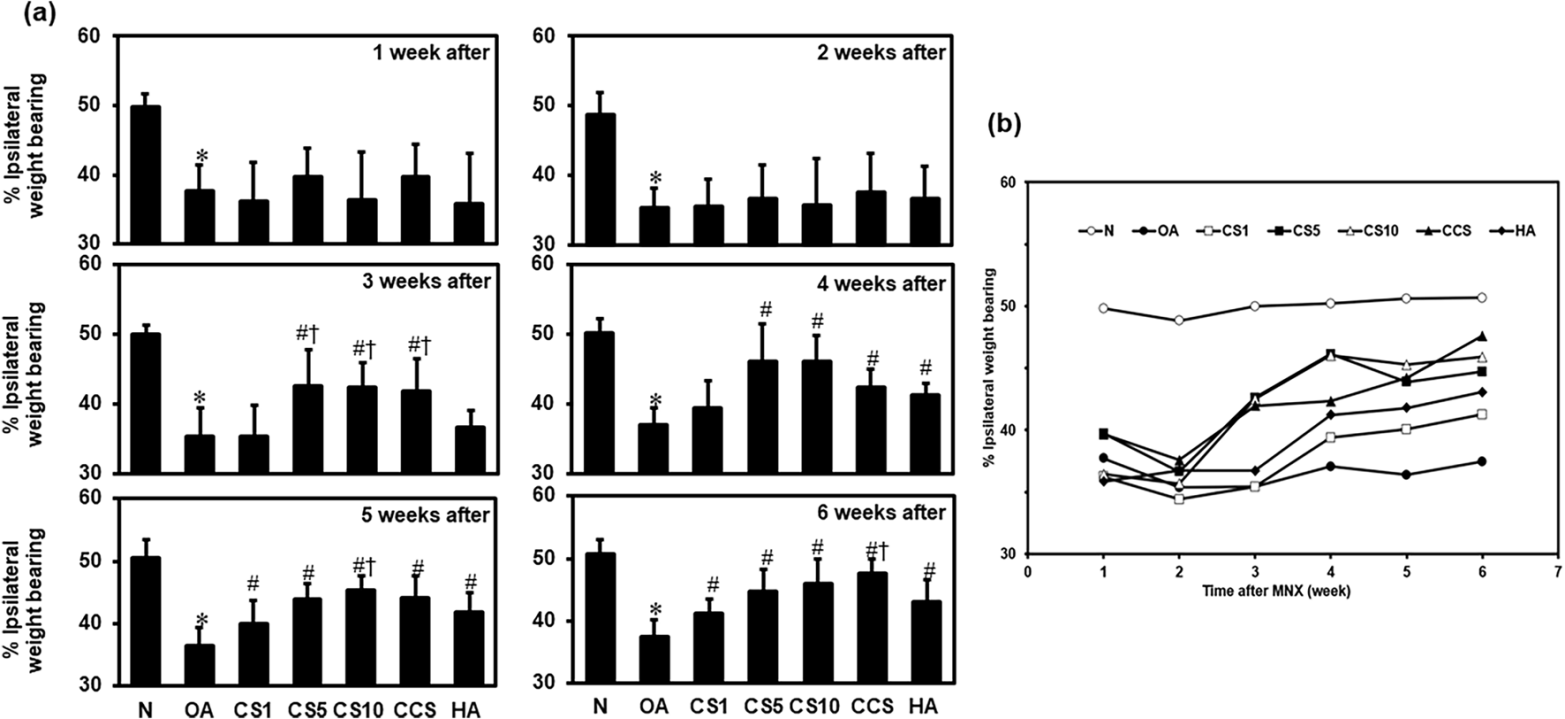
Effects of the Col II solution on knee pain in MNX-induced osteoarthritis in rats. Rats were divided into eight groups (*n* = 6). N group, Normal control; OA group, MNX alone; CS1 group, MNX + type II collagen (1 mg/ml); CS5 group, MNX + type II collagen (5 mg/ml); CS10 group, MNX + type II collagen (10 mg/ml); CCS group, MNX + cartilage + type II collagen; and HA group, MNX + Synvice-One Hyaln G-F 20 (8 mg/ml). Data are expressed as mean ± SD (*n* = 6). *p < 0.05 compared with N group; #*p* < 0.05 compared with OA group; †*p* < 0.05 compared with HA group (*n* = 6)

The OA pain behaviour was evaluated for 6 weeks after MNX surgery by using an in-capacitance meter. At the end of the study period, rats weighed on both hind limbs (50.6% ± 2.2%) in the normal group (N group). The weight distribution on the MNX-operated leg in the OA group (37.4% ± 2.7%) was significantly lower compared with that in the N group (*p* < 0.0001). The weight-bearing of the MNX-induced leg in CS1 group (41.2% ± 2.2%), CS5 group (44.7% ± 3.6%), CS10 group (45.9% ± 4.1%), CCS group (47.6% ± 2.4%), and HA group (43.1% ± 3.5%) were significantly increased compared to OA group (Fig. 2). (Fig. 2a, b).

### Efficacy of Col II solution and hyaluronic acid on structural changes

**Fig. 3:**
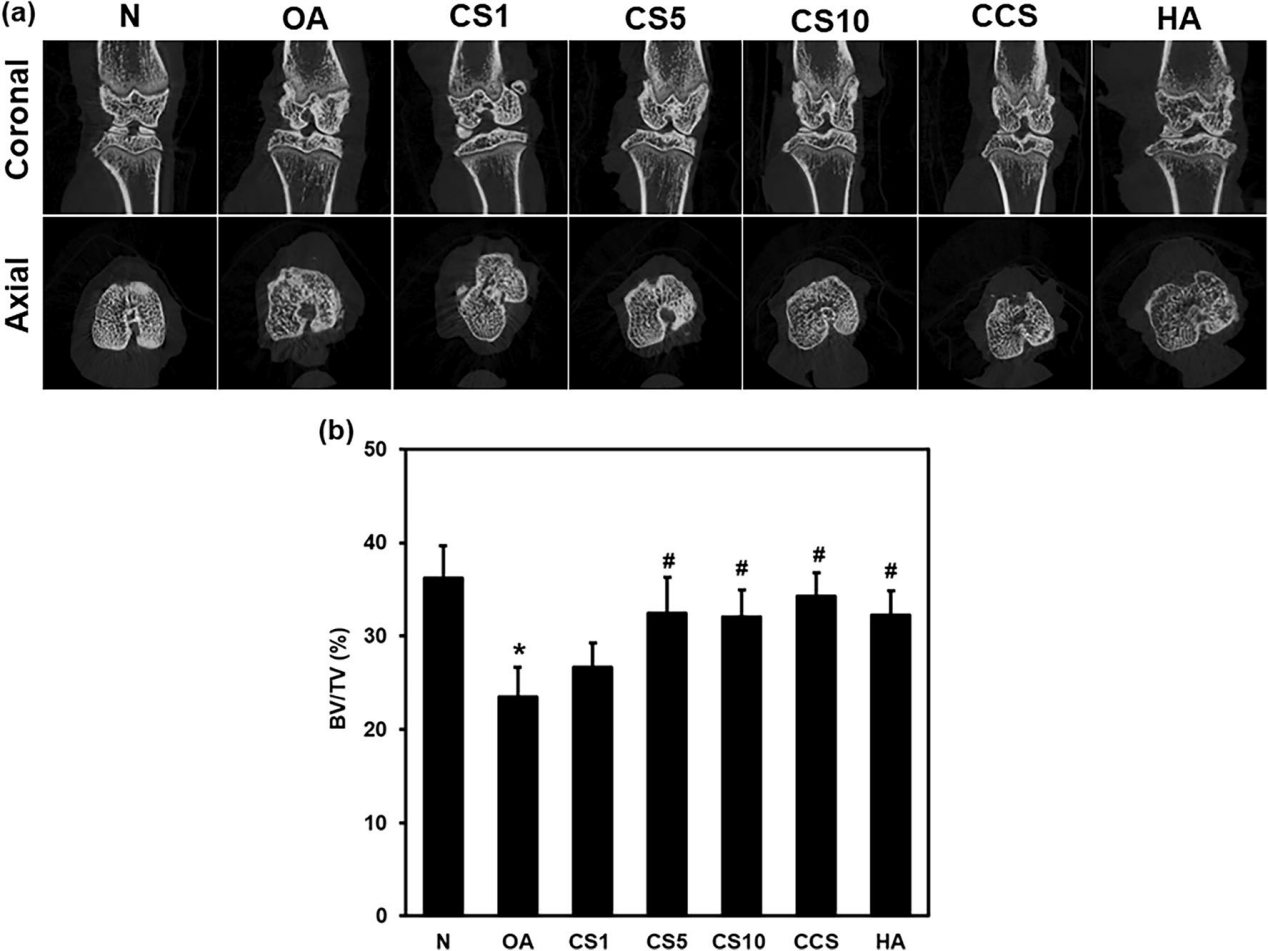
Effects of the Col II solution on cartilage and bone damage in MNX-induced osteoarthritis in rats by micro-CT image. Micro-CT images and coronal sagittal and axial images (**a**), bone volume (**b**) of the hind knee joint of sham and MNX-treated legs were generated by micro-CT 6 weeks after treatments. Data are expressed as mean ± SD (*n* = 6). The differences between treatments with different letters are statistically significant (*p* < 0.05)

The architectural integrity of the knee joint was examined by using a micro-CT machine 6 weeks after MNX surgery. X-ray, coronal and axial micro-CT images showed that the joints had a regular appearance in the N group. The OA group showed a significant (*p* < 0.01) decrease in the bone volume/tissue volume (BV/TV) fraction compared to the normal (N) group. Severe joint destruction was observed in the MNX-induced legs in the OA group. The joint destruction levels of bony surfaces were attenuated in CS1, CS5, CS10, CCS, and HA groups, with a significant (*p* < 0.001) increase in bone volume compared to the OA group (Fig. 3a, b).

### Efficacy of Col II solution and hyaluronic acid on histological change

**Fig. 4:**
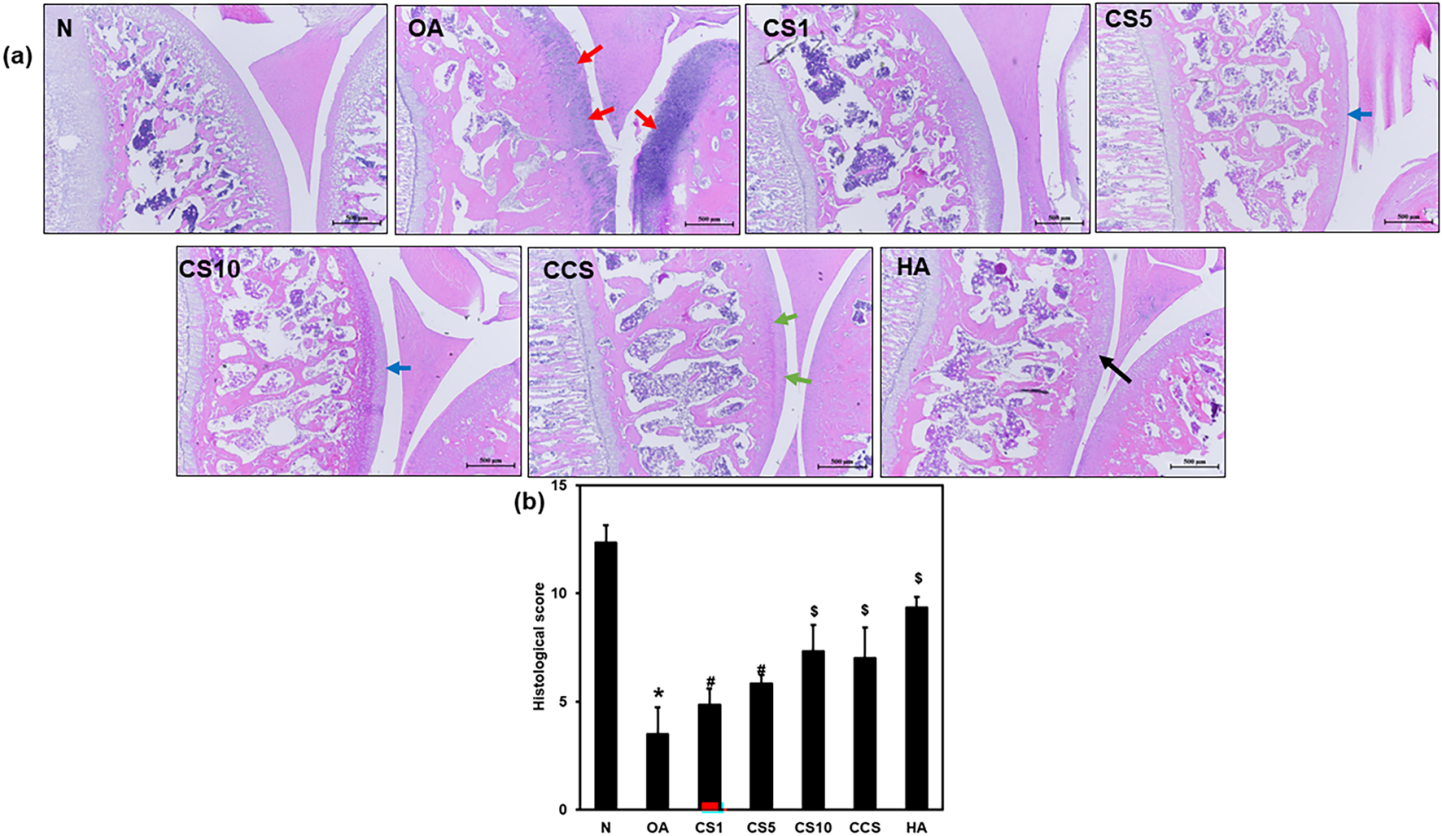
Effects of the Col II solution on cartilage and bone damage in MNX-induced osteoarthritis in rats by histological analysis and scoring for cartilage repair in rat knee joints. The pathological changes in rat knee joints were examined by H&E stain (**a**), scoring (**b**) 6 weeks after MNX treatment (magnification: × 40). The red colour arrow indicates cartilage damage. The blue colour arrow indicates mild cartilage damage. The green and black colour arrow indicates cartilage damage preservation. Data are means ± standard deviation (SD) (*n* = 6). *p < 0.05 compared with N Group; #*p* < 0.05 compared with OA group; ##*p* < 0.001 compared with OA group

**Fig. 5:**
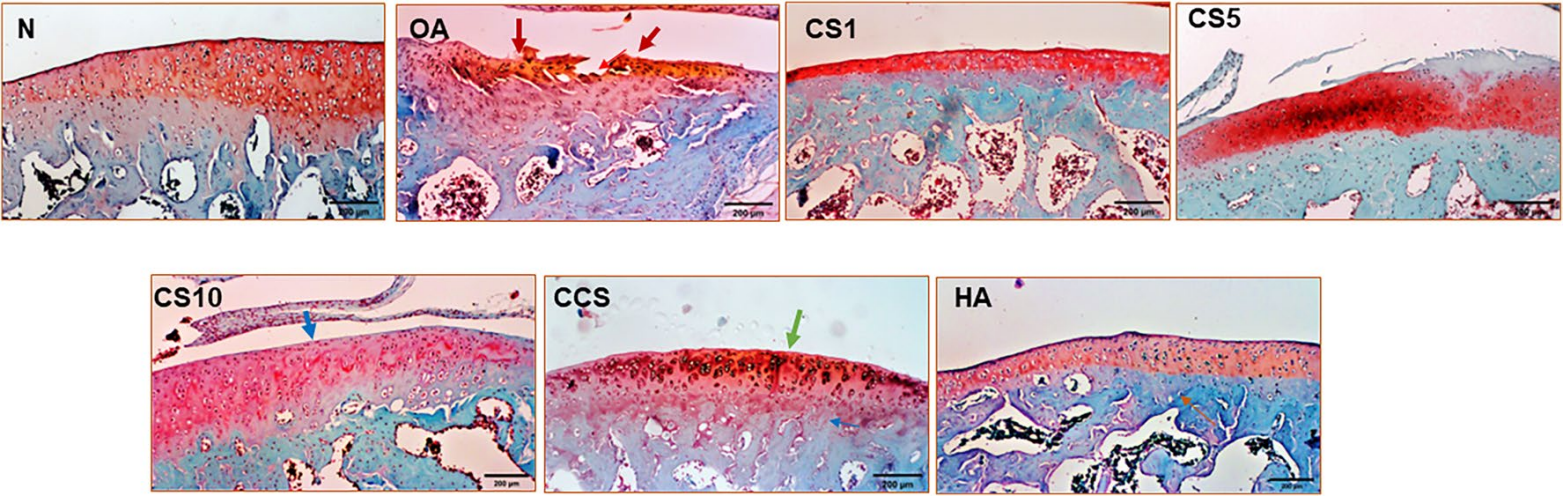
Effects of the Col II solution on cartilage and bone damage in MNX-induced osteoarthritis in rats by safranin-O stain histological analysis for cartilage repair in rat knee joints. The histological and pathological changes in rat knee joints were examined by safranin-O stain 6 weeks after MNX treatment (magnification: × 40). The red colour arrow indicates cartilage damage. The blue colour arrow indicates mild cartilage damage. The green colour arrow indicates cartilage damage preservation

The knee joint histopathological alterations were determined by scoring the H&E and safranin-O staining slides. The histological score was significantly decreased in the OA group compared with that in the N group (3.5 ± 1.2 vs. 12.3 ± 0.8) (*p* < 0.001). The MNX-induced OA joints depicted the loss of cartilage matrix on the surface and overall decreased width of cartilage. Conversely, the histological scores in CS1 (4.8 ± 0.7), CS5 (5.8 ± 0.4), CS10 (7.3 ± 1.2), CCS (7.0 ± 1.4), and HA (9.3 ± 0.5) groups were significantly increased compared with OA group (Fig. 4a, b). The Col II solution-treated knee joints depicted protection of the cartilage matrix on the surface and the width of cartilage was conserved (Fig. 5). The MNX-induced OA knee joints displayed the formation of osteophytes, however, treatment with Col II solution and HA decreased the formation of osteophytes in the knee joints of the MNX-induced OA rats.

### Efficacy of Col II solution and hyaluronic acid on the expression of type II collagen

**Fig. 6:**
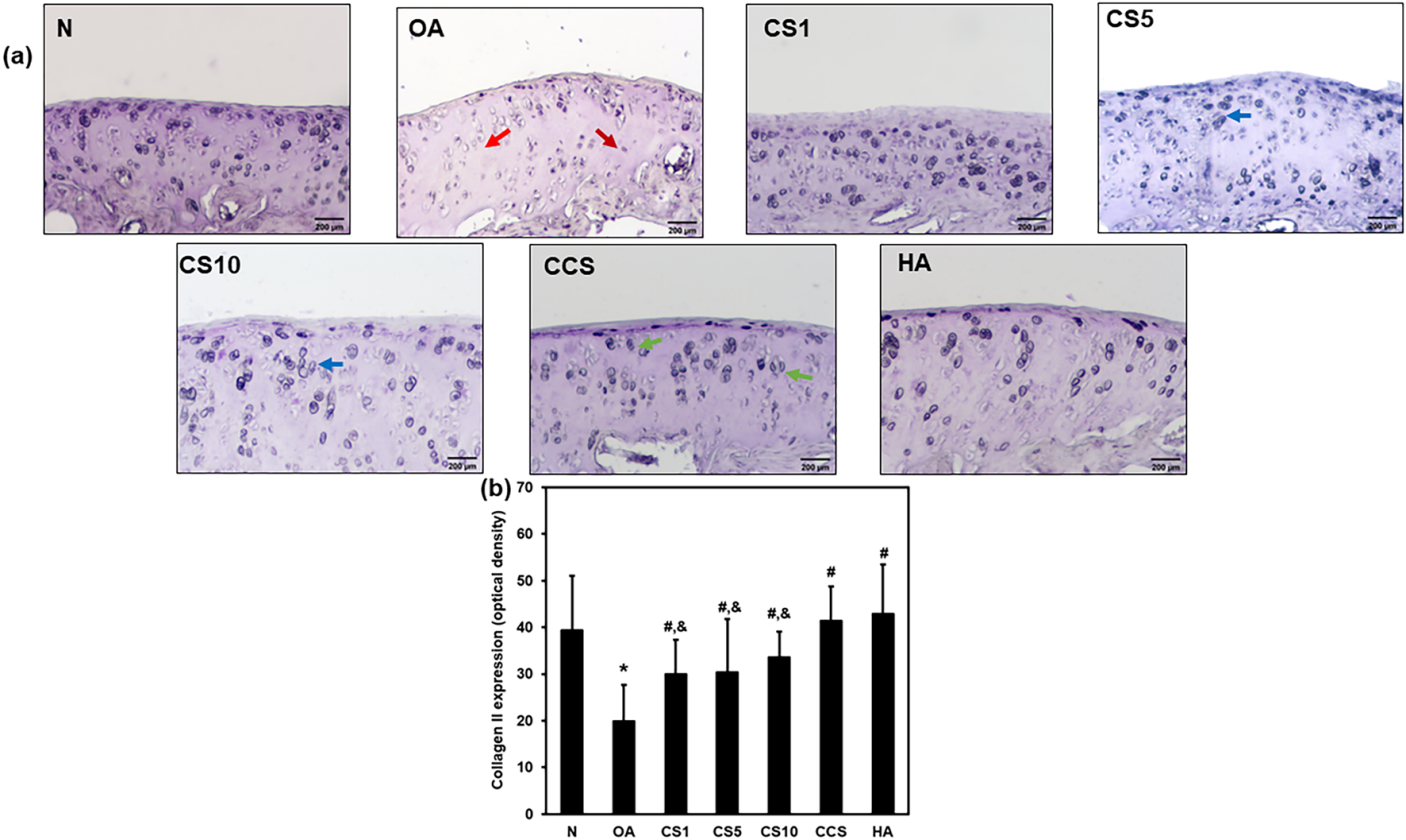
Effect of Col II solution on cartilage collagen II expressions in MNX-induced rat OA. Immunostaining of collagen II expression (**a**), the quantification optical density of collagen II expression (**b**). Data are expressed as mean ± SD. *p < 0.05 compared with N group; #*p* < 0.05 compared with OA group; †*p* < 0.05 compared with HA group (*N* = 6)

To evaluate the regenerative efficacy of Col II solution and hyaluronic acid in MNX-induced OA, the type II collagen expression of chondrocytes was studied. Type II collagen expression density of chondrocytes was significantly decreased (*p* < 0.001) in the OA group compared to that in the N group (19.8 ± 7.8 vs. 39.4 ± 11.6), indicating loss of chondrocytes and type II collagen in OA. The type II collagen expression density of chondrocytes was significantly increased (*p* < 0.001) in CS1 (39.9 ± 7.4), CS5 (30.3 ± 11.4), CS10 (33.6 ± 5.5), CCS (41.3 ± 7.4), and HA (42.9 ± 10.5) groups were significantly increased compared with OA group (Fig. 6a, b), indicating rebuilding of chondrocytes and type II collagen.

### Efficacy of Col II solution and hyaluronic acid on the expression of aggrecan

**Fig. 7:**
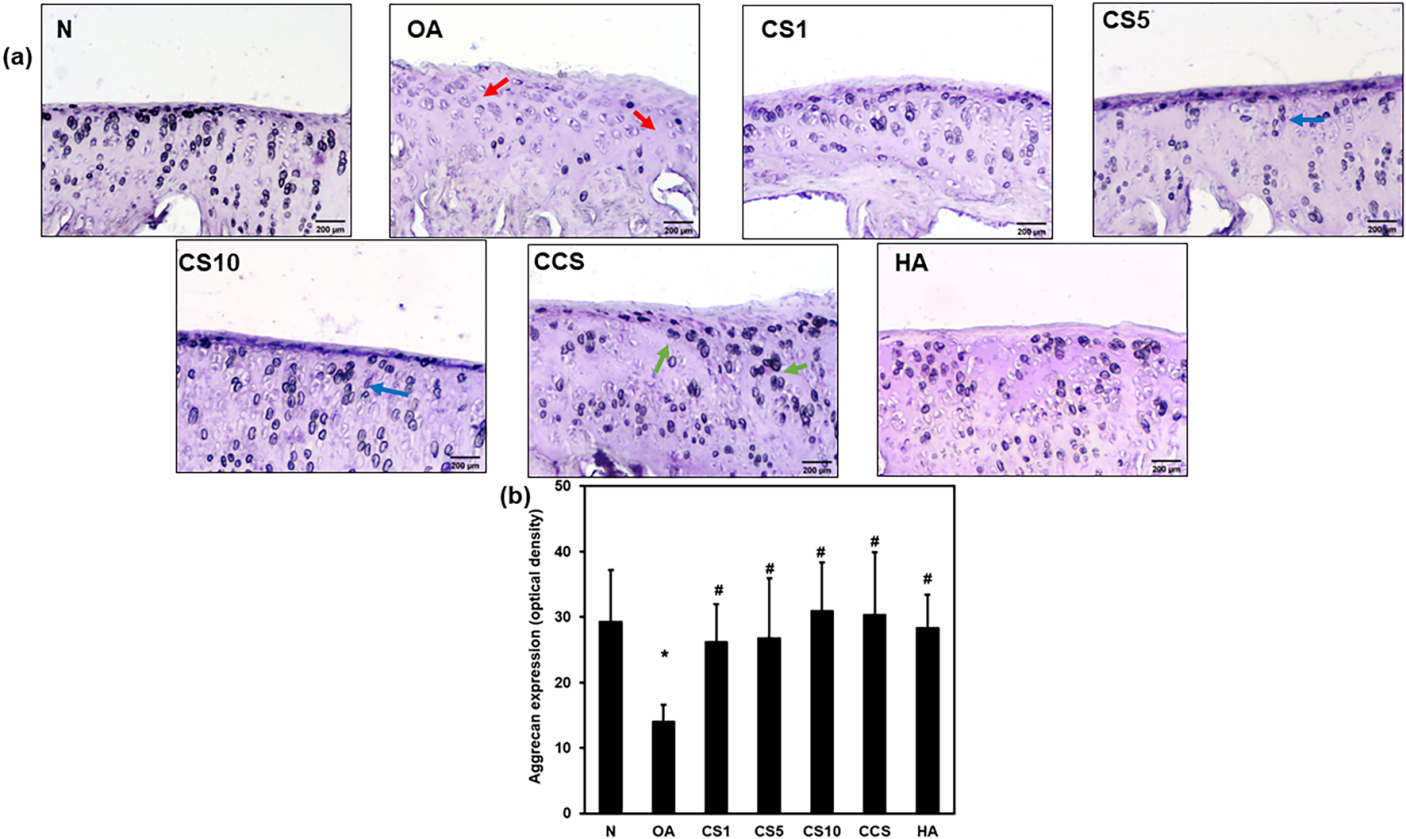
Effects of Col II solution on cartilage aggrecan expression in MNX-induced rat OA. The immunostaining of aggrecan expression (**a**), the quantification optical density of aggrecan expression (**b**). Data are expressed as mean ± SD. *p < 0.05 compared with N group; #*p* < 0.05 compared with OA group (*N* = 6)

The aggrecan expression around the chondrocytes was assessed to evaluate the therapeutic efficacy of Col II solution and hyaluronic acid in the knee of MNX-induced OA. The aggrecan expression around chondrocytes was significantly decreased (*p* < 0.001) in the OA group compared to the normal, N group (14.0 ± 2.5 vs. 19.3 ± 7.9). The Col II solution and hyaluronic acid treatment to OA groups significantly elevated the aggrecan expression as CS1 (26.2 ± 5.8), CS5 (26.7 ± 9.2), CS10 (30.9 ± 7.4), CCS (30.3 ± 9.5), and HA (28.3 ± 5.1) groups were significantly increased compared with OA group (Fig. 7a, b), indicating in response to cartilage injury and the restoration chondrocyte aggrecan with regeneration cartilage in OA.

### Efficacy of Col II solution and hyaluronic acid on the expression of SOX-9

**Fig. 8:**
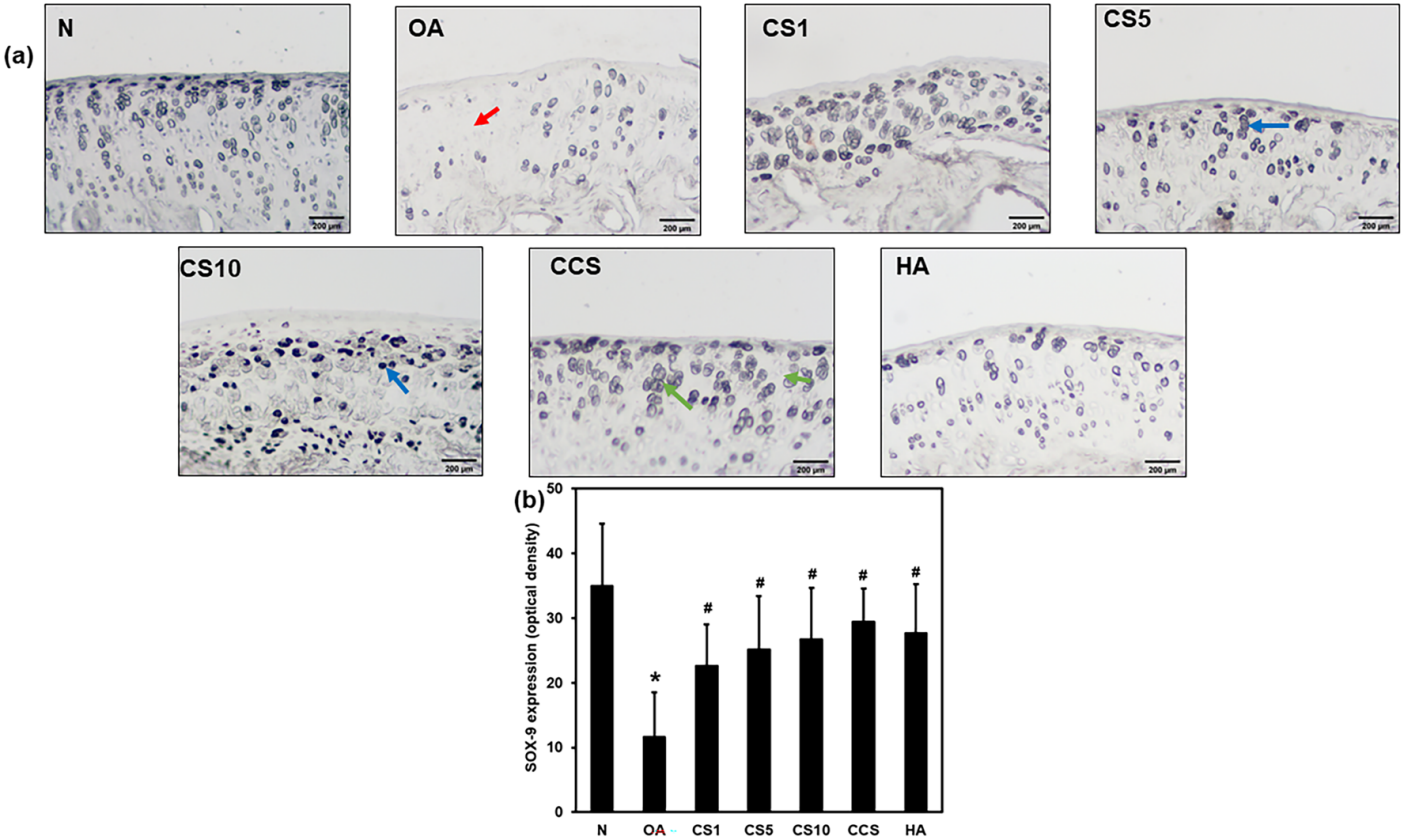
Effects of Col II solution on cartilage SOX9 expression in MNX-induced rat OA. The immunostaining of SOX‐9 expression (**a**), the quantification optical density of SOX‐9 expression (**b**). Data are expressed as mean ± SD. *p < 0.05 compared with N group; #*p* < 0.05 compared with OA group; †*p* < 0.05 compared with HA group (*n* = 6)

The transcription factor, SOX-9 expression in chondrocytes was assessed to study the therapeutic role of Col II and hyaluronic acid in the knee of MNX-induced OA. The SOX-9 expression in chondrocytes was significantly (*p* < 0.001) decreased in group 2 (OA) relative to that of the normal N group (11.6 ± 6.9 vs. 35.0 ± 3.9). The Col II solution and hyaluronic acid treatment to OA groups significantly elevated the aggrecan expression as in CS1 (22.6 ± 6.3), CS5 (25.1 ± 8.3), CS10 (26.7 ± 8.1), CCS (29.4 ± 5.1), and HA (27.6 ± 7.6) groups were significantly increased compared with OA group (Figure. 8b), indicating in response to cartilage injury and the restoration of chondrocytes with regeneration cartilage in OA (Fig. 8a, b).

## Discussion

The conventional methods used for type II collagen extractions are detergents such as Sodium Dodecyl Sulfate (SDS), Triton X-100, Sodium Lauryl Sulfate (SLS) along with crowding agents PEG, dextran sulfate, bovine pancreatic trypsin inhibitor, ribonuclease A, lysozyme, lactoglobulin, haemoglobin, bovine serum albumin, ficoll, poly(sodium 4-styrene sulfonate). In most cases, they also use chemical modifiers anhydride of succinic acid, glutaric acid, phthalic acid, itaconic acid, citraconic acid and maleic acid. These chemical traces were found to be in the final product causing adverse immune-related side effects which are reported in humans (Kroesen et al. 2000).

The advantages of the SCCO_2_ acellular technology include its high efficiency in cellular component removal, no structural damage on the intact ECM scaffold, and no solvent or chemicals needed in the process. Therefore, the final acellular product is free from chemical traces, which is essential in the regeneration without immune-related issues. In addition, the SCCO2 technique also revealed bactericidal and viral inactivation effects, which gives the processed biomaterials a higher degree of safety and biocompatibility (Hsieh and Srinivasan 2020). The product produced using SCCO2 acellular technology preserves the native ECM structure, which helps these products to have good biocompatibility with the potential chemotactic migration of endothelial progenitor cells subsequently enhancing the regeneration (Sung et al. 2023).

An estimated 240 million persons worldwide have symptomatic, activity-limiting OA (Hawker 2016). Approximately 30% of patients more than 45 years old have knee OA demonstrated by radiography, and about 50% have knee symptoms (Jordan et al. 2007). History of joint trauma, including anterior cruciate ligament rupture and ankle fracture, elevates the risk, accounting for 12% of knee OA cases (Brown et al. 2006). The prevalence of symptomatic, radiographic knee OA was 18.7% in women and 13.5% in men (Jordan et al. 2007; Katz et al. 2021). Women have more severe radiographic findings and symptoms as compared to males with OA (Srikanth et al. 2005). OA leads to significant cost and mortality. Of 54 million individuals in the US, 43% experience arthritis-related restrictions in day-to-day activities (Barbour et al. 2017). OA leads to income losses of 65 billion USD and exceeds 100 billion USD in direct medical costs (Hawker 2016; Katz et al. 2021). Knee OA patients spend on average, around in discounted rate of 15,000 USD in their lifetimes on direct medical costs (Losina et al. 2013). OA is frequently connected with comorbidities, which may be due to lack of physical activity, medication toxicity, and the effects of inflammatory cytokines (Hawker 2016; Katz et al. 2021). In the present study, we studied a PTOA rat model to evaluate the efficacy of Col II solution attenuated pain similar to commercial viscosupplementation made up of chemically modified hyaluronic acid that is used as a predicate, this investigation can be extrapolated to human patients.

In the present study, the therapy with Col II solution decreases the pain in a dose and time-dependent means. The destruction of cartilage damage is often connected to increased OA incidence. Trauma and sports injuries cause cartilage destruction subsequently leading to post‐traumatic cartilage injury. The present clinical scenario’s most suited approach and clinical interest are to treat focal cartilage damages to eradicate symptomatic joint pain and evade the development of OA (Chu et al. 2017; Tawonsawatruk et al. 2018). OA is the secondary consequence of accidents causing damage to the joint cartilage and injury, leading to PTOA (Amin et al. 2017; Ross et al. 2017; Tawonsawatruk et al. 2018). Joint pain, swelling, stiffness and crackling sound, with loss of knee joint function are the most general clinical symptoms of OA affecting the quality of life in patients (Wysocka‐Skurska et al. 2016). In the present study, the Col II solution administration decreased the pain in the OA-induced animals thereby maintaining the knee joint function. The most common and significant clinical symptom of knee injury is pain which can be quantified in both animals and humans. The measurement of pain might be beneficial and appropriate to assess the clinical conditions of OA in the rat model (Malfait et al. 2013). The hind leg weight‐bearing asymmetry is used as an additional gauge of motionless knee pain and results in a good and reproducible measurement (Tawonsawatruk et al. 2018). In the current investigation, the pains start to attenuate from the 3rd week and almost subside at the end of the study period in the Col II solution-treated OA rat relative to the HA-treated rats, indicating the Col II solutions therapeutic efficacy in alleviating the pain caused by OA. The probable role of Col II in attenuating OA is through synovitis which is linked to inhibiting the knee joint deterioration and OA pain, thereby it also supplements with type II collagen for the regeneration of cartilage.

In the present study, as evidenced by Micro‐CT the Col II solution injection attenuated the cartilage damage in the knee thereby protecting the bone surface and sustaining the bone volume in MNX-induced OA. Damaged cartilage is imaged using Micro‐CT which offers overall architectural evidence and accurate volumetric calculation of the subchondral bone in the disease progression and therapy of OA. The rapid development of OA was achieved in the surgical induction of OA (Wu et al. 2021). In OA cartilage damage is the primary step, whereas the alterations in the subchondral bone are the ultimate cause of OA (Yu et al. 2013). In the present study, the Col II solution attenuated cartilage damage in OA thereby preventing the alterations in the subchondral bone. The deterioration of the superimposing cartilage is caused by the weak subchondral bone, whereas a good and strong subchondral bone prevents cartilage deterioration (Cox et al. 2011; Yu et al. 2013), thus demonstrating the subchondral bones nature that influences the reliability of articular cartilage in the OA pathogenesis (Yu et al. 2013). In the present study, Col II solution-treated OA attenuated bone volume loss, indicating the Col II solution role on cartilage preservation thereby it attenuated the modifications in the subchondral bone underneath the cartilage, thus attenuating the OA.

The MNX-induced OA leads to cartilage destruction relatively faster than other OA models due to its joint instability. In MNX-induced OA cartilage damage, osteophyte formation, and fibroblast proliferation in the synovium are very similar to human OA. In addition, the MNX-induced OA model is the most suitable for therapeutic intervention in OA (Chu et al. 2017). The inflammation and angiogenesis mainly contribute to the structural modification in OA. Therefore, inhibiting the inflammation and angiogenesis eventually attenuates the structural modifications in the MNX-induced OA (Mapp et al. 2013). In human OA subsets the knee joint injury continues after the inflammatory pain flare reduces, in which chronic synovitis with brief events of inflammation and pain might lead to long-term joint injury (Neogi et al. 2016). In the present study, Col II solution-treated OA attenuated the pain, indicating the Col II solution role in structural cartilage preservation thereby attenuating the inflammation and pain in OA. In the present study, Col II solution-treated OA attenuated inflammation, angiogenesis and osteophyte formation indicating the Col II solution's role in cartilage preservation and regeneration.

In the current investigation, Col II solution-treated OA attenuated by preserving the density of chondrocyte, quality and thickness of cartilage and maintaining the injured cartilage surface. In the MNX OA model, increased inflammation leads to the exacerbation of cartilage damage and osteophyte maturity. Osteophytes are linked to the sensory nerves, which are the important origin of pain in OA. The starting point of the osteophytes is from the periosteum covering bone at the cartilage bone junction. The inflammation in OA is the driving factor for osteoporosis. In addition, osteophyte maturation is the major consequence of angiogenesis. Inflammation-linked angiogenesis may facilitate structural modifications in OA (Wang et al. 2018; Ashraf et al. 2018). The cartilage is a continuous smooth surface, signifying regeneration of the injured cartilage, with no cloning of chondrocytes. The knee joint histological changes were assessed by the cartilage structure, cartilage distribution of glycosaminoglycan and chondrocyte features using a histological grading system (Kim et al. 2018; Wu et al. 2021).

In the current investigation, Col II solution-treated OA attenuated the loss of type II collagen expression thereby preserving the chondrocyte density and cartilage. The OA knee joint the irregular mechanical stress due to stretching by osteophyte and plateau cartilage. The load-bearing transverse force transduced by excessively grown osteophytes indicates catabolic alterations. The major component of the cartilage matrix, type II collagen is decreased in chondrocytes in the middle of distorted osteophytes, which inhibits the repair process (Huebner et al. 2010; Chu et al. 2017). Type II collagen and sulfated proteoglycan in the articular cartilage ECM play a vital role in regulating chondrocyte functions thereby allowing cell‐matrix interactions (Xu et al. 2014). In the present study, Col II solution-treated OA depicted an increased expression of type II collagen that regulates the chondrocyte function leading to allowing cell‐matrix interactions indicating the Col II solution’s role in cartilage regeneration. Type II collagen fragments intravenous injection was known to induce auto‐immune osteoarthritis in the animal model (Proffen et al. 2016). Consequently, the role of ECM in preventing the development of PTOA might seem counter‐intuitive (Proffen et al. 2016; Wu et al. 2021). In the current study, Col II solution-treated OA attenuated the loss of type II collagen expression thereby preserving the chondrocyte density and cartilage.

In the present study, treatment of Col II solution increased the aggrecan and SOX-9 expression thereby preventing the extensive collagen destructions of chondrocytes in articular cartilage. Aggrecan is expressed by chondrocytes and plays a vital role in enabling chondrocyte‐chondrocyte and chondrocyte‐matrix interactions (Kiani et al. 2002; Wu et al. 2021). In the early phases of OA, the therapeutic interventions that enhance aggrecan synthesis can attenuate collagen damage (Roughley & Mort 2014; Wu et al. 2021), demonstrating increased aggrecan synthesis as a defensive apparatus against OA (Ouyang et al. 2019; Wu et al. 2021). SOX-9 plays a vital role in cartilage development via chondrogenesis by activating cartilage-specific genes including type II collagen and aggrecan. In humans, SOX-9 is known to play a significant function in conserving the chondrocyte phenotype in normal and osteoarthritic cartilage. Therefore, decreased expression of SOX-9 in OA leads to cartilage pathology (Orfanidou et al. 2009). The MNX-induced OA, the production of aggrecan and type II collagen by chondrocytes is prevented in the knee joint articular cartilage (Yan et al. 2020). Increased SOX9 expression decreased cartilage damage and subchondral bone plate thickness and inhibited synovitis (Ouyang et al. 2019; Wu et al. 2021). In the present study, treatment of Col II solution increased the aggrecan that acted as a defensive mechanism against OA and SOX-9 expression decreased cartilage damage thereby preventing the extensive collagen destructions of chondrocytes in articular cartilage of MNX‐induced OA rats.

The treatment of OA and the side effects of long-term HA injections are reported as prolonged pain, warmth, and swelling in the injected knee within 24 h of injection and remaining for up to 3 weeks in 27% of patients (Kocak et al. 2018). Pseudogout is also reported with HA injections demonstrated by calcium pyrophosphate crystals on synovial fluid analysis and calcification of cartilage (Kroesen et al. 2000). Crystal-negative arthritis due to granulomatous synovitis with epithelioid histiocytes and multinucleated giant cells resulted in HA injections (Michou et al. 2004). Pseudosepsis with complications that are characterized by severe pain for one to 3 days with massive cellular joint effusion after HA injection, which needs clinical intervention (Conduah et al. 2009). However, the Col II solution is extracted from the supercritical carbon dioxide decellularized porcine cartilage (dPCG), containing native cartilage collagen scaffold ECM, which is rich in type II collagen. The biocompatibility of dPCG was extensively studied over 91 days, which proved nontoxic by assessing the haematology assay, clinical chemistry assay, and blood clotting assay of dPCG implanted animals (Wu et al. 2021). The Col II solution was extracted from the dPCG. Therefore, the Col II solution is nontoxic and biocompatible in rats.

The prospects and significance of therapeutic Col II is to treat incurable OA. OA is incurable since at present no medication is available which can stop or reverse cartilage damage and bone loss. Therefore, added research is being focused on evaluating the various knee joint components that are affected and their role in the contribution to OA pathogenesis. Oncoming in the next few years, many prospective therapies will be directed at cartilage metabolism, inflammation, and subchondral bone remodelling which are forecasted to transform the OA therapy. The injectable form of Col II was manufactured using SCCO_2_ without any chemical traces and will be a suitable candidate for stopping or reversing cartilage damage and bone loss. The probable mechanism of action Col II and dPCG on OA might be acting as a competitive substrate for the cartilage catabolizing agents, metallomatrix proteinase in the articular cartilage. Thus the injected Col II and dPCG protect the extracellular matrix destruction of the knee articular cartilage. The Col II and dPCG offer the scaffold for chondrocytes in the articular cavity to attach, proliferate and repair cartilage damage. The Col II and dPCG encourage aggrecan synthesis and prevent degradation, thereby preventing early stages of OA before widespread collagen destruction. The Col II and dPCG express SOX9 which maintains normal cartilage formation and prevents chondrocyte hypertrophy (Wu et al. 2021).

## Conclusion

Intraarticular Col II solutions showed therapeutic efficacy and cartilage regenerative efficacy in MNX-induced OA rats, similar to HA. In the current study, Col II solution-treated OA attenuated the loss of type II collagen expression thereby preserving the chondrocyte density and cartilage similar to HA. Treatment of Col II solution and HA increased the aggrecan that acted as a defensive mechanism against OA and SOX-9 expression decreased cartilage damage thereby preventing the extensive collagen destructions of chondrocytes in articular cartilage of MNX‐induced OA rats similar to HA.

### Supplementary Information

**Additional file 1:**Experimental protocol of the MNX‐induced OA and the treatment schedule of different groups. The body weight changes were recorded 6 weeks after MNX surgery in rats. Data are means ± standard deviation (SD) (*n* = 6)

## Data Availability

The datasets used and/or analysed during the current study are available from the corresponding author on reasonable request.
