# Supplementary figures and images for: Collagen type II solution extracted from supercritical carbon dioxide decellularized porcine cartilage: regenerative efficacy on post-traumatic osteoarthritis model
Source: Bioresour Bioprocess. 2024 Feb 3;11(1):21. doi: 10.1186/s40643-024-00731-1 (PMC10992551; doi:10.1186/s40643-024-00731-1)

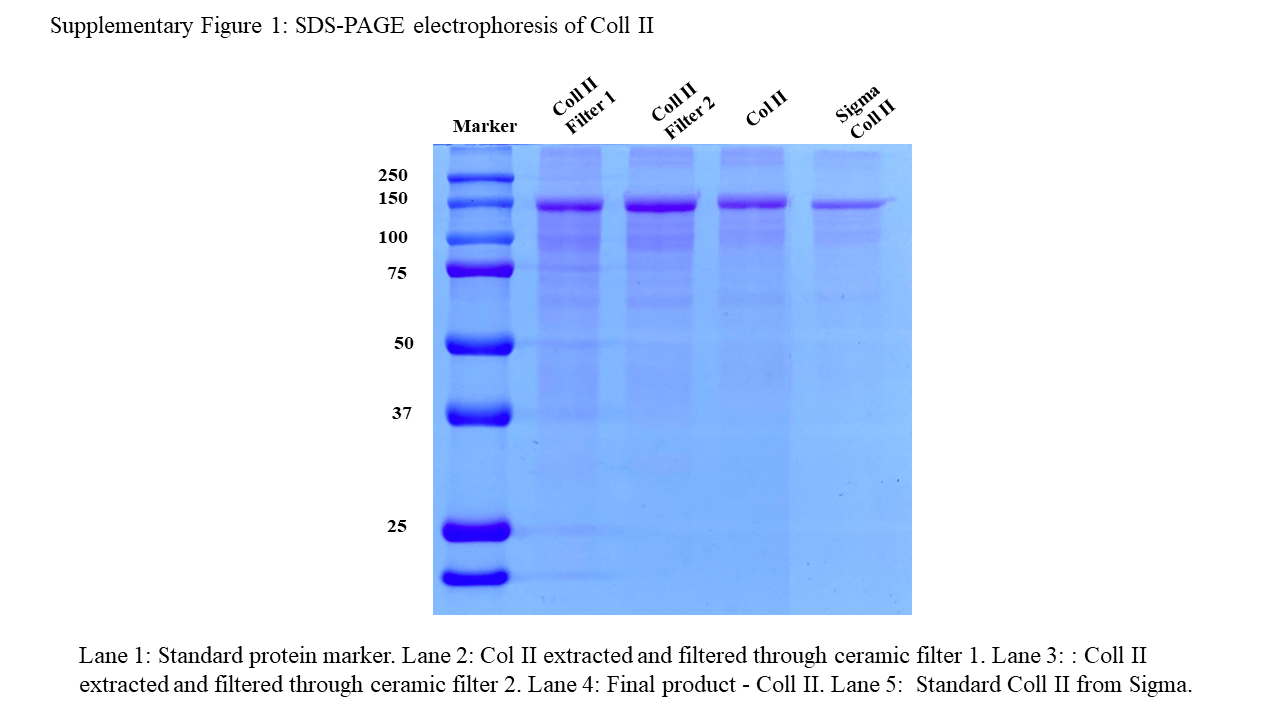

Supplement: Supplementary file 1 — Additional file 1:Experimental protocol of the MNX‐induced OA and the treatment schedule of different groups. The body weight changes were recorded 6 weeks after MNX surgery in rats. Data are means ± standard deviation (SD) (n = 6) [file 40643_2024_731_MOESM1_ESM.tif]
